# Modern Pro-Health Applications of Medicinal Mushrooms: Insights into the *Polyporaceae* Family, with a Focus on *Cerrena unicolor*

**DOI:** 10.3390/molecules30204089

**Published:** 2025-10-15

**Authors:** Dominika Pigoń-Zając, Teresa Małecka-Massalska, Jacek Łapiński, Monika Prendecka-Wróbel

**Affiliations:** 1 Department of Human Physiology of the Chair of Preclinical Sciences, Medical University of Lublin, Radziwiłłowska 11, 20-080 Lublin, Poland; teresa.malecka-massalska@umlub.edu.pl (T.M.-M.); monika.prendecka-wrobel@umlub.edu.pl (M.P.-W.); 2 Faculty of Natural and Technical Sciences, The John Paul II Catholic University of Lublin, Konstantynów 1H, 20-708 Lublin, Poland; jalap@kul.lublin.pl

**Keywords:** *Polyporaceae* family, medicinal mushrooms, *Cerrena unicolor*, health-promoting properties, anticancer activity

## Abstract

Fungal-derived bioactive compounds are emerging as key components in functional food development, offering new opportunities for health-promoting formulations. The *Polyporaceae* family, particularly *Cerrena unicolor*, has demonstrated significant potential due to its rich biochemical profile and diverse health benefits. Despite its extensive bioactive properties, its application in food science and biotechnology remains underutilized. This review explores the bioactive composition, technological potential, and functional applications of *C. unicolor* in innovative food systems. We analyze its antioxidant, antimicrobial, and anticancer effects, focusing on its interactions with dairy-based matrices to enhance bioavailability and therapeutic potential. *C. unicolor* is a valuable source of polysaccharides, phenolics, and enzymatic compounds with antioxidant and anti-inflammatory properties. Its anticancer potential, especially when incorporated into dairy fermentations, opens new avenues for oncology-focused functional foods. Strong antimicrobial activity suggests its potential as a natural biopreservative or bioactive food additive. Bioactive fractions contribute to metabolic health improvements (diabetes management) and tissue regeneration, highlighting their role in next-generation nutraceuticals. Incorporating *C. unicolor* into functional food systems represents a cutting-edge approach to biotechnology-driven health solutions. Further research is required to optimize its formulation, improve bioavailability, and explore regulatory pathways for market implementation.

## 1. Introduction

**Figure 1:**
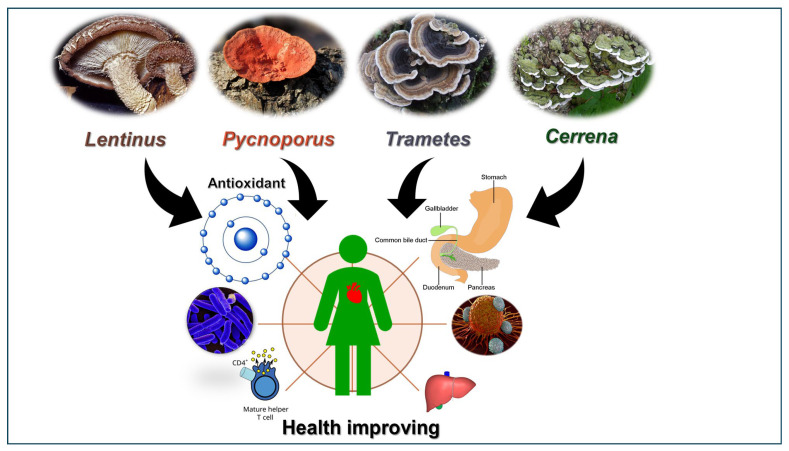
Health-improving activities of selected *Polyporaceae* family mushrooms (own elaboration).

Nowadays, people are exposed to many factors that cause health deterioration and lifestyle diseases, such as diabetes, hypertension, diseases of the respiratory and digestive systems, atherosclerosis, obesity, and some cancers. For this reason, new drugs and increasingly natural compounds are constantly being sought to enhance their biological activity and increase overall human health. Mushrooms, which were originally respected for their culinary and nutritional benefits, are now more and more valued for their numerous significant medicinal properties, to the point where they are used not only as functional foods, but also in the form of nutraceuticals, dietary supplements and mycotherapy products [1,2]. This may be influenced by the fact that medicinal mushrooms have been used in Eastern medicine for centuries to treat a variety of diseases, including cancer [3]. Mushrooms have a crucial role in the natural environment and the human economy. About 700 species of fungus were discovered to have medicinal features, making them a potential source of biologically active chemicals for use in the pharmaceutical industry. In addition to their health-promoting characteristics, arboreal fungi are an exceedingly fascinating source of bioactive substances. These include, e.g., antibiotics with antibacterial and antifungal activity; sterols, flavonoids, tocopherols, carotenoids, phenolic compounds, and indole compounds showing antioxidant properties. Moreover, different compounds from mushrooms such as glycoproteins, glucans, sesquiterpenoids, and triterpenoids exhibit anticancer and immunostimulatory activity, while chitin, statins, and chitosan possess anti-atherosclerosis properties [4,5]. However, mushrooms can also be a source of compounds with different activities such as fungal ribotoxins that are highly selective rRNA ribonucleases (EC 4.6.1.23), which cleave a phosphodiester bond on the ricin–sarcin loop (SRL) of 28S rRNA. Recently, cytotoxic ribonucleases were discovered in the edible fruiting bodies of several *Basidiomycetes*. These proteins, known as ribotoxins or ribotoxin-like proteins (RL-Ps), share the same enzymatic function but have distinct sequences and structures. RL-Ps and ribotoxins may have insecticidal and fungicidal properties. Considering their translation-inhibiting and apoptotic properties, ribotoxins have mostly been employed in medicine as the toxic component of immunotoxins, which are toxins coupled to an antibody directed against a specific target, often an antigen from a tumor cell [6]. The pharmacological actions of a medicinal fungi are largely determined by *in vitro* experiments using cell cultures, which are followed by *in vivo* research in animal models, revealing the enormous potential of mushrooms, fungal extracts, or chemical compounds isolated from them (Figure 1).

The aim of this work was to characterize the modern bioactive substances and health-promoting properties of medicinal mushrooms of the *Polyporaceae* family. Based on the latest scientific research, the health-promoting properties of mushrooms were analyzed, with particular emphasis on bioactive fractions derived from *Cerrena unicolor*.

## 2. Traditional and Current Utilization of the Health-Promoting Properties of Selected Fungi from the *Polyporaceae* Family

### 2.1. The Polyporaceae Family

*Polyporaceae* fungi are widespread in nature and have long played a significant role in traditional Chinese medicine. *Polypores* have a complicated macrostructure, with the flesh made up of various types of hyphae. The rough texture and long life of these fruit bodies are due to strengthening and binding hyphae. The family is one of convenience, with individuals that resemble one another in appearance and grow on wood. The majority of species in this family have hymenium (fertile layer) in vertical holes on the underside of their caps, but some have gills or gill-like structures. Many species are brackets, while others have defined stipes. A considerable body of studies has demonstrated that *Polyporaceae* polysaccharides have a major impact on health [7,8].

### 2.2. The Lentinus

*Lentinus* is a fungal genus belonging to the *Polyporaceae* family. It is widespread, with numerous species occurring in subtropical climates [9].

One of the representative species is *Lentinus edodes*, also known as shiitake.

This medicinal fungus contains B vitamins and minerals, as well as functional metabolites such as lectins, polysaccharides, polysaccharopeptides, and secondary metabolites with bioactivity, such as lentinan and β-(1-3)-glucan with immunomodulatory activity. *L. edodes* extracts and pure chemicals have anticancer, antioxidant, antibacterial, antifungal, cytostatic, and immunomodulatory properties. This mushroom also contains two enzymes capable of inhibiting protein synthesis *in vitro*: edodin and ledodin, both of which exhibit N-glycosylase activity [6,10]. Because of these qualities, a variety of shiitake-derived products are available as supplements [11].

*Lentinus tigrinus* is another interesting wild fungus from the *Polyporaceae* family that is not yet commercially cultivated. It is endemic to southeastern Asia but is also found in Eurasian and northern temperate regions [12]. Its metabolites have shown biological activities, such as the antioxidant activity of ethyl acetate and n-hexane extracts [13], the antiviral activity of laccase [14], and the antibacterial activity of n-hexane and ethanolic extracts [15]. Moreover, the anticancer activity of exopolysaccharides [16] and n-hexane extracts [17,18] has been demonstrated.

The other potentially pro-health mushroom is *Lentinus tuberregium*, a typical tropical wood-rot fungus found in tropical regions and especially recognized for its therapeutic properties in Malaysia and Africa. It was previously used in herbal preparations to treat coughs, indigestion, dysentery, and diarrhea, especially among children [19].

Another example of medicinal mushroom is *Lentinus squarrosulus*, which is commonly employed in African traditional medicines to treat heart problems and mumps. In the work of Mossebo et al., it was shown that the mushroom extract, in various concentrations had antibacterial effects against *Staphylococcus aureus*, *Escherichia coli*, and *Salmonella typhi* [20].

### 2.3. The Pycnoporus

*Pycnoporus* is the next cosmopolitan group of white-rot fungi belonging to the *Polyporaceae* family. The most widely recognized species are *Pycnoporus sanguineus*, *Pycnoporus cinnabarinus*, *Pycnoporus coccineus*, and *Pycnoporus puniceus*. It has been shown that *Pycnoporus* pigments contain cinnabarin, cinnabarinic acid, and tramesanguin, with several proven bioactivities, such as antioxidative activity, free radicals scavenging, antifungal, immunomodulatory, anticancer, larvicidal, leishmanicidal, antiviral, antibacterial, and anti-inflammatory properties [21].

*Pycnoporus sanguineus* is often used in northern Gabon for treating painful menstruation. *P. sanguineus* has been applied in Africa and South America to cure a variety of ailments, including skin lesions, arthritis, sore throat, fever, gout, ulcer, dental discomfort, and hemorrhage [22,23]. It inhibits alpha-amylase and alpha-glucosidase, which can reduce blood glucose levels, insulin resistance, and the risk of developing diabetes complications. In addition, *P. sanguineus* also exhibits antibacterial, antifungal, anti-radical, antiviral, and cytotoxic properties. It has also been shown [24] that *P. sanguineus* extracts demonstrate anti-inflammatory efficacy, with the aqueous extract outperforming niflumic acid but falling short of diclofenac. The crude dichloromethane–methanol extract of *P. sanguineus* outperformed the fraction and doxorubicin in terms of cytotoxicity and anticancer activity across the three cell lines examined. However, the observed cytotoxicity was not limited to cancer cells; normal cells were more susceptible than cancer cells. Other results obtained by [25] suggest that the extracellular fluid isolated from *P. sanguineus*, containing low-molecular-weight secondary metabolite subfractions (ex-LMS) may be an effective source of antioxidative and antibacterial compounds.

*Pycnoporus cinnabarinus*, also known as polypore cinnabar, is a saprophytic white fungus. It can be found in many places in the world. These mushrooms are high in vitamins, minerals, and protein and are widely used in food and medicine. Saprophytes are typically found in soil, urbanized fields, planted trees, and along roadsides. Cinnabarine was also proven to have antiviral and antibacterial properties against dangerous bacteria like *Klebsiella pneumoniae* and *Salmonella typhi* [26]. Other studies confirm that *P. cinnabarinus* demonstrated high antibacterial and bactericidal effects on *Escherichia coli* and *Staphylococcus aureus* and effectively kills *Diatraea magnifactella* larvae [27].

### 2.4. The Trametes

The genus *Trametes* was established in 1835 by Fries. It is part of the medically recognized *Polyporaceae* family, Representatives of the genus are extensively spread across various biotas around the world and have been used in Chinese medicine for several thousand years [28,29]. The medicinal species primarily belong to the genera *Trametes* contain a variety of bioactive components with high and low molecular weights that contribute to their pharmacological potential [30].

*Trametes* species, including *Trametes versicolor* (synonym *Coriolus versicolor*), *Trametes gibbosa*, *Trametes hirsuta*, *Trametes lactinea*, and *Trametes robiniophila* have many pro-health properties especially anticancer potential in the treatment of many different types of cancer. As demonstrated, polysaccharides were considered to be primarily responsible for this anticancer activity [31].

One of the most thoroughly studied species is the “turkey tail” mushroom, *T. versicolor* (L.) Lloyd, which commonly grows on several woody plant species [32]. The mushroom is thought to be beneficial in a variety of ways, including healing, improving organ function, and increasing energy. Among the verified biological activities, antioxidant, antidiabetic, antibacterial, and anti-inflammatory effects are the most frequently indicated, though several studies have also exhibited antiviral, anticancer, and immunomodulatory (as immune-enhancing) activities. The aforementioned pro-health activities have been investigated using different extracts of *T. versicolor* [28,33]. Moreover, anticancer activity has been proven against several cancers such as human hepatocellular carcinoma, human breast adenocarcinoma [34], lung cancer, cervical cancer, and colon cancer [35].

*T. robiniophila* Murr, often known as Huaier, is a sandy beige mushroom that has been employed in traditional Chinese medicine for more than 1600 years. The most frequent medicinal solutions for *T*. *robiniophila* are water-based extracts and granules. The main active component of this mushroom is proteoglycan, which is composed of polysaccharides, amino acids, and water. Research suggests that *T*. *robiniophila* has a significant therapeutic effect on tuberous sclerosis, nephrosis, colitis, and cancers [36]. An example would be a research that showed induction of cancer cell apoptosis in breast cancer [37], lung cancer [38], hepatocellular carcinoma [39,40], and melanoma [41].

Other investigations have shown that fungus *T. robiniophila* reduces the proliferation of human osteosarcoma tumor cells by leading to apoptosis [42,43,44].

Another example of a mushroom used in Asian medicine is *T. gibbosa*, commonly known as “unequal bracket”. *T. gibbosa.* It has been shown to exhibit anticancer, immunomodulatory, antiviral, anti-inflammatory, antioxidant, antibacterial, and neuroprotective properties. Polysaccharides, steroids, and phenolics are known to be responsible for a wide range of their biological functions [45,46,47].

A different mushroom, *T. hirsuta*, popularly branded as a white-rot fungi, is widely distributed around the world. The mushroom’s hairy crown seems pale and gray. *T. hirsuta* is a prospective source of bioactive substances having pharmacological, antioxidant, antibacterial, and antispasmodic properties. Research has shown that *T. hirsuta* has significant analgesic action, indicating its potential as a pain treatment. Its anti-inflammatory and antipyretic qualities also make it a promising therapy for several inflammatory and fever-related conditions [48].

*T. lactinea* (Berk.) Pat, a polypore fungus found in old wood, exhibited wood-rotting activity. Scientists have shown that purified and described heteropolysaccharides may offer an antiproliferation effect in human hepatoblastoma HepG-2 and may serve as a useful alternative therapy for cancer prevention [49].

Additionally, species already extensively described in the literature are as follows: *Ganoderma lucidum*, *Hericium erinaceus*, and *Grifola frondosa*, have a variety of biological properties, especially those that promote health. Polysaccharides found in *G. lucidum* have been proven in modern studies to have anticancer, immunomodulatory, antioxidant, antibacterial, antidiabetic, cardioprotective, and anti-inflammatory properties [50,51] *G. frondosa* polysaccharides have strong anticancer and antiviral activities, as well as the ability to boost the immune system [52]. *H. erinaceus* exhibits notable anti-inflammatory, antioxidant, and antibacterial properties. *H. erinaceus*’ chemical composition contains polysaccharides, terpenoids, and phenolic compounds, all of which have strong antioxidant properties and stimulate endogenous antioxidant enzymes. Furthermore, *H. erinaceus* exhibits remarkable antibacterial activity against bacterial and fungal pathogens, suggesting potential uses in the treatment of antibiotic-resistant diseases. The mushroom’s ability to promote nerve growth factor (NGF) production has demonstrated its promise for preventing and treating neurodegenerative disorders such as Alzheimer’s and Parkinson’s diseases [53].

As indicated by the above-mentioned data, members of the *Polyporaceae* family are abundant in various bioactive compounds with health-promoting activities, as summarized in Table 1.

## 3. *Cerrena unicolor* and the Modern Examples of Its Usage as a Pro-Health Material

### 3.1. Characteristic of Cerrena unicolor

**Figure 2:**
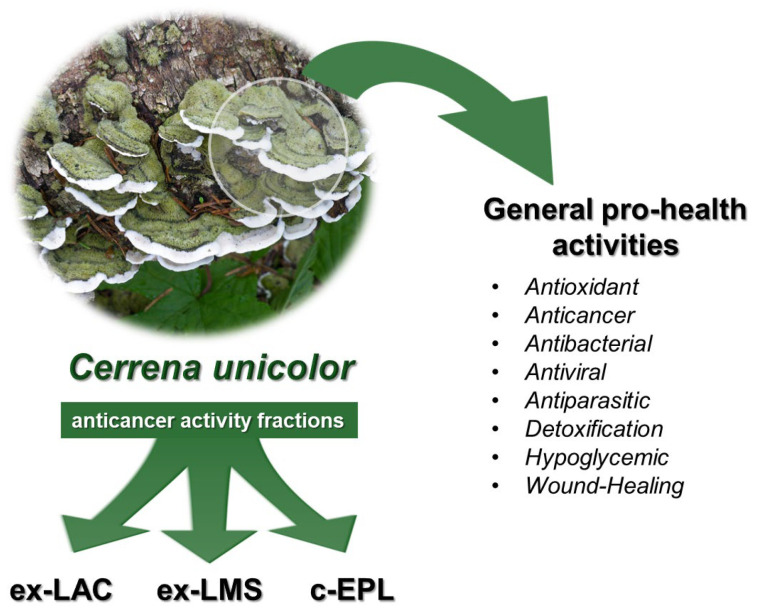
Pro-health activities of bioactive fractions isolated from *C. unicolor* (own elaboration).

One of the main research interests of the last decade is *Cerrena unicolor*, which is a wood-degrading *Basidiomycete* from the *Polyporaceae* family, often known as the mossy maze polypore, and the causal agent of widespread white rot [55]. In its morphological features, it is characterized by a surface-centered zone, hirsute, coated in tufts of hyphae, to nearly glabrous, gray to brown, often green from algae. The hymenophore is milky to whitish, then turns gray and finally brown. Pores are irregular, daedaleoid to labyrinthine, 2–3(4) per millimeter, with thick, tomentose dissepiments. The context (from Latin, the part of the fruiting body supporting the hymenophore) is duplex, up to 3 mm thick, with a pale brownish and corky base layer and a soft, spongy, darker upper half divided by a thin black zone. The sporocarps of this species have a striking look due to their green algae coating. *C. unicolor* is a fungus that shares many features with the *Trametes* genus fungi, with a hirsute surface, irpiciform hymenophore, and a black line between cortex and tomentum. The tube layer is up to 1 cm thick and the same color as the lower layer of the context. Basidiospores are cylindric to ellipsoid, colorless, smooth, measuring 5–7 × 2.5–4 µm [56]. *Cerrena* is an aggressive wood-decaying organism that can occur throughout Europe, Africa, and South America. This fungus is assumed to be parasitic on healthy trees before turning saprobic on dead wood. *C. unicolor* can be found year-round in overlapping clusters on deciduous hardwoods like *Fraxinus excelsior*, *Aesculus hippocastanum*, *Betula* sp., *Acer* sp., *Quercus* sp.,or *Fagus* sp.,but seldom on conifers [57,58]. White-rot fungi uses a variety hydrolytic and oxidative enzymes, including cellulases, xylanases, lignin peroxidase, manganese peroxidase, and laccase, to degrade wood tissue [59,60]. *C. unicolor* has been an extensive subject of scientific research as well as our interest over the last decade. In the next paragraphs, we present many pro-health activities of this mushroom and its extracts (Figure 2).

### 3.2. Antioxidant Activity of Cerrena unicolor

The therapeutic and antioxidant properties of medicinal mushrooms supplement their nutritional value. Free radicals have an essential role in many pathogenic processes in the human body, promoting the development of what are usually referred to as civilization illnesses. They are especially well-known for their impact on oxidative damage to fundamental biomolecules, contributing to aging, cardiovascular disease, cancer, immune system dysfunction, and inflammation. Due to this fact, fungi have also been used as functional food, i.e., they demonstrated positive effects on health in addition to the presence of nutrients usually deemed important in them [5,61]. The white-rot *Basidiomycota*’s life cycle is linked to elevated levels of reactive oxygen species (ROS), which may trigger secondary wood cell wall deterioration. These species have a powerful antioxidative system that includes both enzymatic (laccase, peroxidases, catalase, and superoxide dismutase) and nonenzymatic (phenolic derivatives or polysaccharides) [62]. Laccase (EC 1.10.3.2) is a multicopper-containing oxidase that can oxidize both phenolic and non-phenolic substrates while converting molecular oxygen to water [63].

Studies confirming the antioxidant potential of the fungus *C. unicolor* involved extracting mushroom samples using methanol, ethanol, and dichloromethane in a Soxhlet system. The extracts were tested for total oxidant status (TOS), total antioxidant status (TAS), and oxidative stress index (OSI). The study found that *C. unicolor* had higher TAS and TOS values compared to previously tested *F. pinicola*, *O. olearius*, and *T. terreum* mushrooms’, which suggests a higher antioxidant potential [64].

Another research presenting antioxidant properties of three low-molecular-weight subfractions (ex-LMS I-III) derived from *C. unicolor* using three tests: the free radical 1,1-diphenyl-2-picryl-hydrazyl (DPPH)-scavenging test, the [2,2′-azinobis-(3-ethylbenzothiazoline-6-sulfonic acid)] (ABTS) radical-scavenging test, and the hydroxyl radical-scavenging activity assay (OH). Results indicated that ex-LMS III had the highest DPPH radical-scavenging activity, followed by ex-LMS I and ex-LMS II. The ABTS test shows that ex-LMS fractions, particularly ex-LMS III, had slightly decreased scavenging capability compared to the DPPH technique. The order of hydroxyl radical-scavenging activity is determined by comparing IC_50_ values, with ex-LMS III having the highest value [65].

In another research, idiophasic cultures of the white-rot fungus *C. unicolor*, generated three bioactive fractions: extracellular laccase (ex-LAC), endopolysaccharides (c-EPL), and low-molecular-weight subfractions (ex-LMS). The ex-LMS fraction showed the strongest reducing capability, while the scavenging abilities of *C. unicolor* c-EPL were lower but still significant. Chemiluminescent technique measured the EC_50_ values for ex-LMS, trolox, and ascorbic acid, while the EC_50_ values for ABTS and DPPH scavenging techniques were 25.0 and 85.3 µg/mL, respectively. The excellent scavenging activity of ex-LMS suggests it could be a valuable source of naturally derived antioxidants. The ex-LAC probes also showed significant prooxidative potential [62]. The present results confirm the antioxidant capabilities of fractions obtained from the *C. unicolor* mushroom.

In addition, another study [66] investigated the antioxidant properties of low-molecular-weight subfractions obtained from secondary metabolites generated by *C. unicolor*. Research was carried out by the use of the same tests. The research indicates that subfraction 6 had the greatest antioxidant activity. At the same time, the poorest antioxidant capabilities were discovered in the fraction with mass below 1.5 kDa subfraction S and subfraction 3. Another example of investigation of the antioxidant capabilities of *C. unicolor* was demonstrated by Pięt et al. Scientists examined the activity of fungal fractions obtained from milk-supplemented *Cerrena* cultures. Furthermore, cultivating the examined fungus on milk-supplemented media may enhance the health benefits of both milk and mushrooms. The extracts had greater amount of protein, less free amino acids, and more phenolic compounds than milk. They also had a free radical scavenging effect, which could be linked to the elevated activity of catalase and superoxide dismutase (SOD). The zymographic examination revealed significantly higher catalase and SOD activity, as well as a lower number of free radicals than the control. This could indicate a relationship between enzyme activity and free radical concentration [67].

### 3.3. Anticancer Activity of Cerrena unicolor

Cancer is a serious global danger and the second leading cause of mortality, after cardiovascular disorders, in developed countries [68]. Standard treatments for cancer include chemotherapy, surgery, hormone-targeted therapies, radiation, and immunotherapies. These treatments can cause a variety of negative effects, such as pain, fatigue, weakness, immunosuppression, digestive system symptoms, and many other factors that may lead to a deterioration of the patient’s condition during therapy [3]. With this in consideration, researchers are always searching for natural therapeutic approaches that minimize side effects and are safe for patients. Various studies have revealed that medicinal mushrooms have a positive impact on quality of life and reduce the negative effects of conventional medicines [69]. Various extracts from medicinal mushrooms, such as polysaccharides, especially ß-glucans, have been examined *in vivo* and *in vitro*, and in human experiments [70]. Many clinical trials have been undertaken to investigate their complimentary use with various drugs, such as when combined with chemotherapy. The results reveal relevant health advantages such as general disease-free survival in colorectal cancer patients and enhanced comfort of life among lung cancer patients [71].

Most published studies report the anticancer properties of *C. unicolor*. One of the mentioned studies is the research of Matuszewska et al. who hypothesized that ex-LAC may be cytotoxic to leukemic cell lines and primary CLL cells. The XTT test showed a high cytotoxic effect on all tested cell lines and CLL primary cells, with half-maximum inhibitory doses ranging from 0.4 to 1.1 µg/mL. Fluorescence microscopy and SEM analysis also showed apoptotic modifications in Jurkat and RPMI 8226 cells exposed to ex-LAC compared to control cells. The findings were consistent with the flow cytometry of Jurkat cells for apoptosis. In conclusion, *C. unicolor* ex-LAC strongly induces cell death and may be a potential therapeutic agent for hematological neoplasms [72].

Another experiment confirmed the anticancer properties of *C. unicolor* isolates. The cytotoxic and antiproliferative effects of laccase (LAC), endopolysaccharides (c-EPL), and low-molecular-weight (ex-LMS) fractions were investigated for the first time. Antitumor activity was tested on cervical carcinoma cell lines such as SiHa and its metastasis into the small intestine, with HSF performing as a control. The study used the MTT test and microscopic observation to determine the cytotoxic and antiproliferative effects of *C. unicolor* fractions. Results showed that LAC and ex-LMS fractions had a cytotoxic effect against cervical cancer and normal fibroblasts. LAC had a lower ED_50_ for SiHa cells than CaSki cells, while the ED_50_ for HSF was not noticeable. Ex-LMS did not differ significantly between cancer and normal cells. LAC effectively reduced cervical tumor and normal fibroblast cell density, but destruction was only observed in SiHa and CaSki cells, not in HSF control cells [73].

The purpose of the next study was to inquire into the anticancer properties of low-molecular-weight subfractions derived from *C. unicolor.* The tested model consisted of cell lines of human colon cancer cells—HT-29 and normal human colon epithelial cells—CCD 841 CoTr. In this study, it was exhibited that the low-molecular-weight subfractions inhibited human colon cancer cells HT-29. All six extract subfractions reduced cell growth in a dose-dependent manner, with S, 3, 4, and 5 showing the greatest inhibition of HT-29 cell proliferation. Microscopic analysis showed all extracts induced programmed cell death (apoptosis) in HT-29, but apoptosis was observed on a significantly lower level in CCD 841 CoTr cells, with no or minimal necrosis in both cell lines [66].

Another interesting research revealed the anticancer properties of three low-molecular-weight subfractions isolated from secondary metabolites of *C. unicolor.* Cancer cell growth inhibition *in vitro* was tested by the MTT assay. It was found that ex-LMS III had the highest inhibitory activity against breast tumor cells MDA-MB-231, PC3, and MCF7, with a 59.08% inhibition at a 15 μg/mL dosage, while normal fibroblast cells remained unaffected [65].

Nevertheless, additional studies on *C. unicolor*’s biochemical characteristics and anti-tumorigenic activity toward colon cancer cells were investigated. The effect of supplementing the fungal culture medium containing cow’s milk on these activities was also investigated. The toxicity effect data was obtained from two separate assays, NR and MTT. May–Grünwald–Giemsa cells staining further proven these results. Extracts at a concentration of 200 μg/mL substantially decreased the viability of HT-29 and SW948 cancer cell lines, with the greatest effect observed in HT-29 cells. Pro-apoptotic effects toward cancer cells (HT-29, LS 180, and SW948) were also revealed. When compared to the control, the *C. unicolor* 1-II extracts showed considerable pro-apoptotic activity [67].

Subsequent studies aimed to confirm the antitumor properties of the ex-LMS subfraction in relation to the CT-26 colon cancer cell line compared to normal L929 mouse fibroblasts. The research was carried out using the MTT test, which assessed the antiproliferative activity of selected ex-LMS concentrations, and the Electric Cell–Substrate Impedance Sensing (ECIS) technique for monitoring cellular changes in real time. The research showed that the ex-LMS subfractions had a significantly higher antiproliferative effect on CT-26 tumor cells compared to L929 cells. Results obtained from the ECIS assay revealed a substantial decrease in impedance and resistance characteristics in the CT-26 cell line after treatment with various concentrations of ex-LMS compared to untreated and normal cells. In the instance of colon cancer cells, a decrease in impedance and resistance indicates a decrease in cell viability, but the physiological activities of L929 fibroblasts remain unchanged [74].

Other studies confirming anticancer activity fractions isolated from *C. unicolor* focused on two ovarian cancer cell lines, NIH: OVCAR-3 and Caov-3. The first stage of the investigation determined the concentration of laccase (LAC) obtained from *C. unicolor* that reduced growth and proliferation without causing cytotoxicity in the examined cell lines. In the second part of the investigation, the ECIS system was applied to evaluate how the LAC affected cancer cell proliferation and migration. Research has shown that LAC exhibits cytotoxic and antiproliferative effects on ovarian cancer cells from the Caov-3 and NIH: OVCAR-3 lines. Caov-3-line cells demonstrated higher sensitivity to the LAC preparation compared to NIH: OVCAR-3 cells. On the other hand, LAC indicated a milder effect on normal cells of the L929 line than cisplatin, suggesting the possibility for further *in vivo* investigations [75]. Another research was performed with LAC extract derived from the medicinal fungus *C. unicolor* on CT-26 colon cancer cells. Preliminary cytotoxicity studies revealed that the tested extract caused damage to cancer cells but had no meaningful effect on normal L929 cells. The ECIS results showed that the laccase had potential anticancer action against colon cancer cells [76]. However, the antitumor potential of *C. unicolor* is not its only biological action.

### 3.4. Antimicrobial and Antiviral Activity of the Cerrena unicolor

Overuse of commercial antimicrobial medicines for treating infectious diseases has led to multidrug resistance in pathogenic bacteria. In this context, new antibiotics are urgently needed to treat diseases caused by resistant bacteria, especially food-borne pathogens. Many studies have shown that several mushroom components have antimicrobial effects. Cell wall glucans have immunomodulatory characteristics, and mycelium-secreted secondary metabolites were presented to be effective against bacteria and viruses [77]. That is why the study described below, which illustrates the antibacterial and antiviral use of isolates from the *C*. *unicolor* fungus, highlights its importance.

In the research conducted by the team of [62], an attempt was made to demonstrate antibacterial properties of fractions such as c-EPL, ex-LMS, and ex-LAC against marine bacteria *Vibrio fischeri* obtained from *C. uniolor*. The study assessed the cellular activity of *V. fischeri* after exposure to c-EPL, ex-LMS, and ex-LAC enzymes. The antibacterial activity was evaluated using a modified well diffusion test. The results showed that *V. fischeri* had 38% cell damage after 5 min of exposure to ex-LAC enzyme and 51% after 15 min. The ex-LAC sample effectively inhibited E. coli, while *V. fischeri* had 85% cell destruction after 5 min and 88% after 15 min of c-EPL exposure. The c-EPL sample showed antibacterial efficacy against *S. aureus*, while exposing *V. fischeri* to low-molecular-weight extracellular metabolites resulted in 95% cell death after 5 min and 98% after 15 min. The research found that ex-LMS was more effective than ex-LAC and acted similarly to c-EPL on bacterial cells.

In continuation of their research, the team of [65] also examined the antibacterial properties of ex-LMS I, ex-LMS II, and ex-LMS III subfractions. A study on *S. aureus*, *E. coli*, and *Bacillus subtilis* found that ex-LMSI, ex-LMSII, and ex-LMSIII had high antibacterial activity against all Gram-positive and Gram-negative bacteria species tested. *S. aureus* was the most vulnerable, while *B. subtilis* and *E. coli* had the lowest sensitivity. The ex-LMS II fraction was the most efficient against all bacteria tested, with ex-LMS II inhibiting *S. aureus* more effectively than *B. subtilis* or *E. coli.* In the study, researchers suggested that thelow-molecular-weight secondary metabolites in *C. unicolor* culture fluid, particularly ex-LMS II, could be a potential source of antibacterial compounds.

The antimicrobial activity of *C*. *unicolor* extracts is not only limited to antibacterial properties. In the report by [73], three bioactive fractions, laccase (LAC), low-molecular-weight (ex-LMS), and endopolysaccharides (c-EPL), were investigated for their antiviral and immunostimulatory properties for the first time. In this study, LAC antiviral efficacy was demonstrated against *Human Herpes Virus type 1* (HHV-1) and *Encephalomyocarditis virus* (EMCV) in a virus replication suppression experiment. The study found that LAC induces antiviral mechanisms in susceptible cells, reducing virus titers by at least 1.5–2 logs compared to the control group. LAC also showed anti-HHV-1 and anti-EMCV action, but no immediate virucidal activity. A low dose of LAC demonstrated anti-EMCV activity. LAC therapy significantly reduced the cytopathic effect of viruses in sensitive cells, indicating antiviral efficacy. LAC from *C. unicolor* had greater antiviral action against HHV-1 compared to EMCV. The research hypothesized that this activity could occur due to the formation of reactive oxygen species (ROS) by LAC, disrupting cellular structure and inhibiting metabolism after viral infection. The fungal c-EPL fraction also increased TNF-α and IL-6 synthesis and secretion by THP-1-derived macrophages, suggesting its immunostimulatory effect. In another study, extracts from the basidiocarp, submerged-growing mycelia, and crude exopolysaccharide precipitates of *C*. *unicolor* were examined. Submerged mycelium isolates from *C. unicolor* were effective against bacteria and yeasts. These extracts were most effective in inhibiting the growth of *S. aureus*, *Enterococcus faecium,* and *Micrococcus luteus* [78]. However, microorganisms such as bacteria and viruses are not the only factors that could decrease humans health, and fungi might be used more efficiently against different organisms.

### 3.5. Antiparasitic Properties of Cerrena unicolor

Parasitic infections are a very serious and common problem among humans and animals alike in today’s world. Despite significant progress in medicine, they remain common, and new and safe methods of combating them are still being sought. Plants and fungi are well-known for producing natural medicines that can be used to treat a variety of ailments. Many of them are used to treat parasite-related gastrointestinal disorders in both humans and animals. The World Health Organization (WHO) reported that over 1.5 billion people worldwide have been infected with intestinal nematodes in 2018. Abdominal infections caused by parasitic nematodes considerably interfere with the healthy functioning of the human system, and the symptoms of the disease are chronic and especially dangerous in children [79].

Scientists have discovered the antinematode properties of extracellular low-molecular-weight subfractions (ex-LMS) derived from liquid medium for growing idiophasic *C. unicolor* cultures. *Rhabditis nematodes*, which can infect the external auditory canal, gastrointestinal tract, and urinary tract in humans, were used as a study model. Nematodes were cultured *in vitro* and introduced to S1–S6 subfractions, with albendazole as a positive control and 0.6% NaCl and water as solvents. After 24 h, the nematodes were examined using optical and stereoscopic microscopes. Results showed that both the entire fractions and the 0.02–1.5 kDa fraction showed nematicidal activity, reducing the number of live nematodes by 50%. *C. unicolor* fractions showed higher nematicidal characteristics at lower concentrations compared to albendazole. All six subfractions from the 0.02–1.5 kDa fraction decreased nematode viability, with subfraction6 having the strongest nematicidal properties. The study suggests *C. unicolor* as a promising new option for nematode infection treatment [80].

### 3.6. Detoxification Activity of Cerrena unicolor

The antitumor and anti-microorganism activity of *C*. *unicolor* extracts is well described within the literature; however, there are some reports of other pro-health properties of tested compounds isolated from this mushroom. One of them is detoxification. Consuming excessive amounts of alcohol has been linked to liver tissue destruction, as well as a variety of unfavorable metabolic changes. Alcohol-induced liver illnesses are divided into three stages: fatty liver, steatohepatitis, and alcoholic cirrhosis. Due to this fact, researchers are looking for new and natural ways to treat and prevent these conditions. Numerous mushrooms, including *Pleurotus florida*, *G. frondosa*, *L. edodes*, and *Tricholoma lobayense*, and *Agaricus bisporus* were shown to be effective in treating liver diseases caused by various substances. The study presented by [81] examined the histological, biochemical, and cytological effects of EPSs from four different macrofungal species, including *C. unicolor* on a rats alcohol-induced liver model. The authors examined the potential therapeutic benefits of crude exopolysaccharides derived from mushrooms on the indicators of alcoholic liver injury. Alanine transaminase (ALT), lactate dehydrogenase (LDH), and DNA fragmentation levels in serum activity and integrity were assessed after the rats were sacrificed. They revealed that ALT levels were reduced, and the histological and cytological image of treated hepatocytes were like normal tissue (uninjured). This may suggest that mushroom extracts, including those from *C. unicolor*, could protect the liver against alcohol-induced injury.

Aflatoxin B1 (AFB1) is a well-known carcinogen that is highly harmful to human health and the digestive tract. It was revealed that it may be detoxified by laccases, as it is a multicopper oxidase that eliminates toxins and pollutants from the environment. The work [82] discovered a novel laccase isolated from the white-rot fungus *C*. *unicolor* that had the ability to catalyze the degradation of AFB1. The fungal laccase activity toward AFB1 detoxification was expanded by cloning and sequencing the lac-2 cDNA, which encodes a full-length protein of 512 amino acids. The breakdown of AFB1 by Lac-2 was carried out *in vitro*. Moreover, syringaldehyde (SA) and acetosyringone (AS) seemed to be comparable mediators that potently boosted AFB1 breakdown by Lac-2. These results hold promise for the potential usage of Lac-2 as a novel aflatoxin oxidase to detoxicate food contaminated with AFB1.

### 3.7. Hypoglycemic Effect of Cerrena unicolor

The aim of the next study was to investigate the hypoglycemic efficacy of crude exopolysaccharides (EPS) generated by *C*. *unicolor* isolates in diabetic rats induced by streptozotocin. After administration of the EPS preparation to rats, their serum glucose level decreased by 61.23%. Histological observations determined by staining pancreatic tissues revealed that the Langerhans islet areas and number of cells of diabetic rats increased in response to EPS therapy. Finally, the data clearly indicate that EPS generated by *C. unicolor* lowered blood sugar levels in diabetic rats infected with streptozotocin. As a result, the examined mushroom EPS could be developed into promising oral hypoglycemic medicines for diabetes mellitus management. This was the first *in vivo* investigation employing EPS from indigenous mushroom isolates for therapeutic purposes. Moreover, there are no extensive remarks in the literature regarding the antidiabetic activity of extracts obtained from *C*. *unicolor*, providing a potential research area for more comprehensive studies [83].

### 3.8. Wound-Healing Activity of Cerrena unicolor

Wound healing is a complex process crucial for repairing the barrier function of the skin. It can be stopped by different disorders, leading to chronic wounds that pose a significant medical burden. Such wounds do not heal normally and are frequently accompanied by a pro-inflammatory environment caused by elevated proteinases, hypoxia, and accumulation of bacteria. It is essential to create an effective dressing for wounds with antibacterial, anti-inflammatory, and antioxidant capabilities. The use of natural materials for wound healing has been extensively studied, but only a few are currently commercialized or used in healthcare settings. As a result, it is critical to investigate the potential of natural bioactive compounds in skin tissue regeneration. Further investigation is needed to identify novel natural bioactive compounds, their role in wound healing, and their potential as alternatives to conventional antibiotics [84].

Extracts and their metabolites obtained from medicinal mushroom have been proven effective for wound treatment, with contributions to multiple processes, including immunological epithelial cell activation, cytokines, extracellular matrix, growth factors, numerous inflammatory intermediates, and reactive oxygen species [85]. In research presented by [86], the viability of using fibrin glue enhanced with two low-molecular-weight anticancer fractions, S5 and S6, of fungal *C. unicolor* as a viable, sustainable, and safe drug delivery strategy was evaluated for the first time. The two fractions with the most promising effectiveness in suppressing the proliferation of HT 29 tumor cells were used. One of the fractions also had the ability to induce coagulation in human plasma. The physical properties of the generated fibrin clot were examined using thromboelastometry and confocal microscopy. Enriching the obtained results fibrin sealant with S6 and S5 fractions increased its structure and viscoelastic properties. The S5 fraction’s influence on cell proliferation stimulation in the presence of fibrinogen is similarly significant, indicating that it can promote wound healing. Simultaneously, the optimum amounts of components S5 and S6 may inhibit migration, modulating the activity of matrix metalloproteinases. The potential application in the treatment of postoperative wound healing while also reducing potential metastatic processes is incredibly helpful.

### 3.9. Challenges and Future Perspectives

Large-scale production of medicinal mushroom extracts involves many stages and can be considered a rather expensive process by today’s standards. However, the isolation of active extracts from medicinal mushrooms using technologies such as solid-state cultivation (SSC), submerged liquid substrate cultivation, or fed-batch or two-stage cultivation is relatively simple and relies on precipitation with hot water and ethanol. Furthermore, crude extracts are known to exhibit pharmacological activity equal to or greater than purified compounds, suggesting potential synergistic effects of several naturally occurring compounds. Therefore, expensive and highly technical purification procedures for the isolation of pure pharmaceutical substances may not be necessary [87]. Apart from the obvious benefits associated with the use of mushroom extracts in medicine, their toxicological assessment is also necessary, which includes steps such as full identification of the extract’s composition and testing for cytotoxicity or mycotoxin contamination.

One of the disadvantages of previous research on compounds derived from the medicinal fungus *C. unicolor* is that it primarily focused on *in vitro* cell lines. It is critical to continue these research efforts by employing *in vivo* animal trials and including them into translational, preclinical, and clinical studies, which would boost the possibility of commercializing the findings in the future. Another and one of the most important disadvantages of the current study is the lack of investigation into the potential molecular mechanisms of extracts obtained from *C. unicolor*. This research could contribute to improving our understanding of molecular pathways and uncover novel therapeutic targets in anticancer therapy, as well as supplement conventional oncological therapies by minimizing side effects in the future. The mechanistic data of *C. unicolor* extracts are under evaluation at this moment and will provide new knowledge on this topic.

## 4. Conclusions

The complex analysis of the *Polyporaceae* family, with special emphasis on *C. unicolor*, exhibited the fungi’s importance in both traditional medicine and modern therapeutic research. Their utilization as nutritional supplements and natural treatments represent a rich ethnopharmacological past that helps scientists to predict their bioactive components and their pro-health activities. Current studies have proven that *C*. *unicolor* contains a wide range of pharmacologically active compounds. Furthermore, several lines of evidence of its anticancer capabilities, such as the extracts derived from its cultivation in cows’ milk, suggest a prospective application in decreasing cancer cell viability. These findings are also supported by reports of antibacterial, antiviral, and antiparasitic properties, providing proof that *C. unicolor*’s bioactive fractions could be the basis for novel therapeutic tactics. The specific and complex interplay between *C. unicolor*’s bioactive compounds and their several biological activities gives justification for considering them as new, integrated therapeutics. In this regard, *C. unicolor* is not only a model organism for studying natural product pharmacology but also may be a possible foundation for future drug discovery. Despite these encouraging results, significant challenges remain. To fully realize the therapeutic potential of *C. unicolor* and similar species, extraction processes must be standardized, and potential molecular mechanisms of action must be investigated. Future studies should employ modern analytical methods to better understand the interactions of its bioactive components with essential cellular targets. This comprehensive approach will open the path for the development of more effective and specific treatments. In conclusion, the historical use and modern scientific investigation of *C*. *unicolor* exemplify the essence of ethnopharmacology, a discipline that values ancient wisdom while embracing modern innovation. The convergence of these approaches has enormous potential for improving healthcare outcomes and developing the area of natural product-based therapies. Continued multidisciplinary research in this field is critical to turning traditional medicine’s rich past into fresh, evidence-based clinical applications.

## Figures and Tables

**Table 1:** Medicinal properties of compounds isolated from various mushroom species.

Mushroom Species	Bioactive Fraction/Compound	Medicinal Properties	Source
*Lentinus edodes*	lentinan, lectins, β-(1-3)-glucan, ledodin, and edodin	anticancer, antioxidant, antibacterial, antifungal, cytostatic immunomodulatory activities, and rRNA N-glycosylase activity inhibits protein synthesis and leads to cell death	[6,10,11]
*Lentinus tigrinus*	laccase, n-hexane extracts, ethanolic extracts, and exopolysaccharides	antioxidant, antiviral, antibacterial, and anticancer activities	[13,14,15,16,17,18]
*Lentinus tuberregium*	extracts	therapeutic properties to treat coughs, indigestion, dysentery, and diarrhea	[19]
*Lentinus squarrosulus*	extracts	treat heart problems and mumps and antibacterial activities	[20]
*Pycnoporus sanguineus*	crude dichloromethane–methanol extract and low-molecular weight (ex-LMS) metabolites	antibacterial, antifungal, anti-radical, antiviral, and cytotoxic properties and anti-inflammatory activity	[24,25]
*Pycnoporus cinnabarinus*	cinnabarine	antiviral and antibacterial properties and insecticidal potential	[27,54]
*Trametes versicolor*	extracts	antioxidant, antidiabetic, antibacterial, and anti-inflammatory activities	[28,33,34,35]
*Trametes robiniophila*Murr	proteoglycan and polysaccharides	therapeutic effect on tuberous sclerosis nephrosis, colitis, and anticancer activities	[36,37,38,39,40,41]
*Trametes gibbosa*	polysaccharides, steroids, and phenolics	anticancer, immunomodulatory, antiviral, anti-inflammatory, antioxidant, antibacterial, and neuroprotective properties	[45,46,47]
*Trametes hirsuta*	extracts	pharmacological, antioxidant, antibacterial, antispasmodic, anti-inflammatory, and antipyretic activities	[48]
*Trametes lactinea*	heteropolysaccharides	antiproliferation and anticancer activities	[49]

## Data Availability

No new data were created or analyzed in this study. Data sharing is not applicable to this article.

## Abbreviations

The following abbreviations are used in this manuscript:

ABTS	2,2′-azinobis-(3-ethylbenzothiazoline-6-sulfonic acid) radical-scavenging test
AFB1	aflatoxin B1
ALT	alanine transaminase
AS	acetosyringone
c-EPL	endopolysaccharides
DPPH	free radical 1,1-diphenyl-2-picryl-hydrazyl-scavenging test
EC_50_	half maximal effective concentration
ECIS	electric cell–substrate impedance sensing technique
EMCV	encephalomyocarditis virus
EPS	exopolysaccharides
ex-LAC	extracellular laccase
ex-LMS	low-molecular-weight secondary metabolite subfractions
HHV-1	human herpes virus type 1
IC_50-_	half maximal inhibitory concentration
IL-6	interleukin-6
LDH	lactate dehydrogenase
MTT-3	(4,5-dimethylthiazol-2-yl)-2,5-diphenyltetrazolium bromide test
NGF	nerve growth factor
NR	neutral red assay
OH	hydroxyl radical-scavenging activity assay
OSI	oxidative stress index
RL-Ps	ribotoxin-like proteins
ROS	reactive oxygen species
SA	syringaldehyde
SEM	scanning electron microscope
SOD	superoxide dismutase
SRL	ricin–sarcin loop
SSC	solid-state cultivation
TAS	total antioxidant status
TNF	total antioxidant status
TOS	total oxidant status
WHO	World Health Organization
XTT	(2,3-bis-(2-methoxy-4-nitro-5-sulfophenyl)-2H-tetrazolium-5-carboxanilide) test

## References

[1] Venturella G., Ferraro V., Cirlincione F., Gargano M.L. (2021). Medicinal mushrooms: Bioactive compounds, use, and clinical trials. Int. J. Mol. Sci..

[2] Elkhateeb W.A., Elkhateeb W.A. (2020). What Medicinal Mushroom Can Do?. Chem. Res. J..

[3] Jeitler M., Michalsen A., Frings D., Hübner M., Fischer M., Koppold-Liebscher D.A., Murthy V., Kessler C.S. (2020). Significance of Medicinal Mushrooms in Integrative Oncology: A Narrative Review. Front. Pharmacol..

[4] Elsayed E.A., El Enshasy H., Wadaan M.A.M., Aziz R. (2014). Mushrooms: A potential natural source of anti-inflammatory compounds for medical applications. Mediators Inflamm..

[5] Ślusarczyk J., Adamska E., Czerwik-Marcinkowska J. (2021). Fungi and algae as sources of medicinal and other biologically active compounds: A review. Nutrients.

[6] Citores L., Ragucci S., Russo R., Gay C.C., Chambery A., Di Maro A., Iglesias R., Ferreras J.M. (2023). Structural and functional characterization of the cytotoxic protein ledodin, an atypical ribosome-inactivating protein from shiitake mushroom (*Lentinula edodes*). Protein Sci..

[7] Joshi M., Bhargava P., Bhatt M., Kadri S., Shri M., Joshi C.G. (2021). Polyporaceae BT—Mushrooms of Gujarat.

[8] Guo Y., Chen X., Gong P. (2021). Classification, structure and mechanism of antiviral polysaccharides derived from edible and medicinal fungus. Int. J. Biol. Macromol..

[9] Kirk P., Cannon P., Stalpers J., Minter D.W. (2008). Dictionary of the Fungi.

[10] Citores L., Ragucci S., Gay C.C., Russo R., Chambery A., Di Maro A., Iglesias R., Ferreras J.M. (2024). Edodin: A New Type of Toxin from Shiitake Mushroom (*Lentinula edodes*) That Inactivates Mammalian Ribosomes. Toxins.

[11] Gaitán-Hernández R., López-Peña D., Esqueda M., Gutiérrez A. (2019). Review of Bioactive Molecules Production, Biomass, and Basidiomata of Shiitake Culinary-Medicinal Mushrooms, Lentinus edodes (Agaricomycetes). Int. J. Med. Mushrooms.

[12] Seelan J.S.S., Justo A., Nagy L.G., Grand E.A., Redhead S.A., Hibbett D. (2015). Phylogenetic relationships and morphological evolution in Lentinus, Polyporellus and Neofavolus, emphasizing southeastern Asian taxa. Mycologia.

[13] Sadi G., Emsen B., Kaya A., Kocabaş A., Çınar S., Kartal D.İ. (2015). Cytotoxicity of some edible mushrooms extracts over liver hepatocellular carcinoma cells in conjunction with their antioxidant and antibacterial properties. Pharmacogn. Mag..

[14] Xu L., Wang H., Ng T. (2012). A laccase with HIV-1 reverse transcriptase inhibitory activity from the broth of mycelial culture of the mushroom Lentinus tigrinus. J. Biomed. Biotechnol..

[15] Dulay R.M.R., Arenas M.C., Kalaw S.P., Reyes R.G., Cabrera E.C. (2014). Proximate composition and functionality of the culinary-medicinal tiger sawgill mushroom, Lentinus tigrinus (higher Basidiomycetes), from the Philippines. Int. J. Med. Mushrooms.

[16] He P., Wu S., Pan L., Sun S., Mao D., Xu C. (2016). Effect of tween 80 and acetone on the secretion, structure and antioxidant activities of exopolysaccharides from Lentinus tigrinus. Food Technol. Biotechnol..

[17] Ragasa C.Y., Tan M.C.S., De Castro M.E., De Los Reyes M.M., Oyong G.G., Shen C.C. (2018). Sterols from lentinus tigrinus. Pharmacogn. J..

[18] Mohammadnejad S., Pourianfar H.R., Drakhshan A., Jabaleh I., Rezayi M. (2019). Potent antiproliferative and pro-apoptotic effects of a soluble protein fraction from culinary-medicinal mushroom Lentinus tigrinus on cancer cells. J. Food Meas. Charact..

[19] Kumar M., Kaviyarasan V. (2012). Distribution of Lentinus tuberregium (Fr.), an indigenous edible medicinal mushroom in Tamil Nadu, South India. J. Acad. Indus. Res..

[20] Mossebo D.C., Metsebing B.-P., Oba R., Tsigaing Tsigaing F., Ryvarden L., Fonkui T.Y., Mungoh Tata C., Ndinteh D.T. (2020). Comparative evaluation of antifungal and antibacterial activities of crude extracts of Pleurotus sajor-caju, Pleurotus tuber-regium and Lentinus squarrosulus (Basidiomycota, Pleurotaceae, Lentinaceae) from Cameroon. Eur. J. Biol. Biotechnol..

[21] Téllez-Téllez M., Villegas E., Rodríguez A., Acosta-Urdapilleta M.L., O’Donovan A., Díaz-Godínez G. (2016). Mycosphere Essay 11: Fungi of Pycnoporus: Morphological and molecular identification, worldwide distribution and biotechnological potential. Mycosphere.

[22] Bourdette J.O.O., Calixte N.E.H., Misso R.-L.N.M., Obiang C.S., Prudence Y.Y., Ondo J.P., Roger N.A.G., Engonga L.-C.O. (2019). Phytochemical Screening, Antioxidant and Antiangiogenic activities of Daedaleopsis nitida, Pycnoporus sanguineus and Phellinus gilvus Medicinal Mushrooms from Gabon. Pharm. Chem. J..

[23] Mounguengui S., Saha Tchinda J.B., Ndikontar M.K., Dumarçay S., Attéké C., Perrin D., Gelhaye E., Gérardin P. (2016). Total phenolic and lignin contents, phytochemical screening, antioxidant and fungal inhibition properties of the heartwood extractives of ten Congo Basin tree species. Ann. For. Sci..

[24] Bourdette J.O.O., Ndong H.C.E., Bourobou H.P.B., Engonga L.C.O. (2022). Mycochemical analysis, anti-inflammatory and cytotoxic activities of *Pycnoporus sanguineus* (L.) Murrill, a medicinal mushroom from Gabon. World J. Biol. Pharm. Res..

[25] Jaszek M., Osinska-Jaroszuk M., Sulej J., Matuszewska A., Stefaniuk D., Maciag K., Polak J., Matuszewski L., Grzywnowicz K. (2015). Stimulation of the antioxidative and antimicrobial potential of the blood red bracket mushroom Pycnoporus sanguineus (higher Basidiomycetes). Int. J. Med. Mushrooms.

[26] Sharma A., Bhardwaj G., Nayik G.A. (2023). Edible and Medicinal Mushrooms of the Himalayas: Climate Change, Critically Endangered Species, and the Call for Sustainable Development.

[27] Díaz-Godínez G., Téllez-Téllez M., Rodríguez A., Obregón-Barbosa V., De Lourdes Acosta-Urdapilleta M., Villegas E. (2016). Enzymatic, antioxidant, antimicrobial, and insecticidal activities of Pleurotus pulmonarius and Pycnoporus cinnabarinus grown separately in an airlift reactor. BioResources.

[28] Soković M., Glamočlija J., Ćirić A., Petrović J., Stojković D. (2018). Chapter 5- Mushrooms as Sources of Therapeutic Foods. Ther. Foods.

[29] Olou B.A., Krah F.S., Piepenbring M., Yorou N.S., Langer E. (2020). Diversity of *Trametes* (Polyporales, Basidiomycota) in tropical Benin and description of new species *Trametes parvispora*. MycoKeys.

[30] Sharma D., Singh V.P., Singh N.K. (2018). A Review on Phytochemistry and Pharmacology of Medicinal as well as Poisonous Mushrooms. Mini Rev. Med. Chem..

[31] Muñoz-Castiblanco T., Mejia-Giraldo J.C., Puertas-Mejia M.A. (2020). Trametes genus, a source of chemical compounds with anticancer activity in human osteosarcoma: A systematic review. J. Appl. Pharm. Sci..

[32] Tišma M., Žnidaršič-Plazl P., Šelo G., Tolj I., Šperanda M., Bucić-Kojić A., Planinić M. (2021). Trametes versicolor in lignocellulose-based bioeconomy: State of the art, challenges and opportunities. Bioresour. Technol..

[33] Shokrzadeh M., Azdo S., Amirahmadi M., Habibi E. (2017). Anti-diabetic effect of methanol extract of Trametes versicolor on male mice. J. Maz. Univ. Med. Sci..

[34] Janjušević L., Pejin B., Kaišarević S., Gorjanović S., Pastor F., Tešanović K., Karaman M. (2018). *Trametes versicolor* ethanol extract, a promising candidate for health-promoting food supplement. Nat. Prod. Res..

[35] Shnyreva A.V., Shnyreva A.A., Espinoza C., Padrón J.M., Trigos Á. (2018). Antiproliferative Activity and Cytotoxicity of Some Medicinal Wood-Destroying Mushrooms from Russia. Int. J. Med. Mushrooms.

[36] Pan J., Yang C., Jiang Z., Huang J. (2019). Trametes robiniophila murr: A traditional Chinese medicine with potent anti-tumor effects. Cancer Manag. Res..

[37] Wang J., Wang X., Chen T., Jiang L., Yang Q. (2017). Huaier Extract Inhibits Breast Cancer Progression Through a LncRNA-H19/MiR-675-5p Pathway. Cell. Physiol. Biochem..

[38] Chen Y., Wu H., Wang X., Wang C., Gan L., Zhu J., Tong J., Li Z. (2018). Huaier Granule extract inhibit the proliferation and metastasis of lung cancer cells through down-regulation of MTDH, JAK2/STAT3 and MAPK signaling pathways. Biomed. Pharmacother..

[39] Hu Z., Yang A., Su G., Zhao Y., Wang Y., Chai X., Tu P. (2016). Huaier restrains proliferative and invasive potential of human hepatoma SKHEP-1 cells partially through decreased Lamin B1 and elevated NOV. Sci. Rep..

[40] Bao H., Liu P., Jiang K., Zhang X., Xie L., Wang Z., Gong P. (2016). Huaier polysaccharide induces apoptosis in hepatocellular carcinoma cells through p38 MAPK. Oncol. Lett..

[41] Zhang F., Zhang Z., Liu Z. (2013). Effects of Huaier aqueous extract on proliferation and apoptosis in the melanoma cell line A875. Acta Histochem..

[42] Li C., Wu X., Zhang H., Yang G., Hao M., Sheng S., Sun Y., Long J., Hu C., Sun X. (2015). A Huaier polysaccharide inhibits hepatocellular carcinoma growth and metastasis. Tumour Biol..

[43] Zhao X., Ma S., Liu N., Liu J., Wang W. (2015). A polysaccharide from Trametes robiniophila inhibits human osteosarcoma xenograft tumor growth in vivo. Carbohydr. Polym..

[44] Zhao X., Ma S., Liu N., Liu J., Wang W. (2015). A polysaccharide from Trametes robiniophila Murrill induces apoptosis through intrinsic mitochondrial pathway in human osteosarcoma (U-2 OS) cells. Tumour Biol..

[45] Jouybari H.B., Bekhradnia A., Mirzaee F., Hosseinzadeh M.H., Habibi E. (2022). Chemical Composition of the Lumpy Bracket Mushroom (*Trametes gibbosa*). Res. J. Pharmacogn..

[46] Knežević A., Stajić M., Sofrenić I., Stanojković T., Milovanović I., Tešević V., Vukojević J. (2018). Antioxidative, antifungal, cytotoxic and antineurodegenerative activity of selected Trametes species from Serbia. PLoS ONE.

[47] Zengin G., Karanfil A., Uren M.C., Kocak M.S., Sarikurkcu C., Gungor H., Nancy Picot C.M., Mahomoodally M.F. (2016). Phenolic content, antioxidant and enzyme inhibitory capacity of two: *Trametes* species. RSC Adv..

[48] Begum H.A. (2023). Exploring the pharmacological potential of Trametes hirsuta (White Rot Fungi): Analgesic, anti-Inflammatory, antispasmodic and antimicrobial activities. Pure Appl. Biol..

[49] He N., Tian L., Zhai X., Zhang X., Zhao Y. (2018). Composition characterization, antioxidant capacities and anti-proliferative effects of the polysaccharides isolated from *Trametes lactinea* (Berk.) Pat. Int. J. Biol. Macromol..

[50] Xie J., Lin D., Li J., Zhou T., Lin S., Lin Z. (2023). Effects of Ganoderma lucidum polysaccharide peptide ameliorating cyclophosphamide-induced immune dysfunctions based on metabolomics analysis. Front. Nutr..

[51] Ahmad M.F., Alsayegh A.A., Ahmad F.A., Akhtar M.S., Alavudeen S.S., Bantun F., Wahab S., Ahmed A., Ali M., Elbendary E.Y. (2024). Ganoderma lucidum: Insight into antimicrobial and antioxidant properties with development of secondary metabolites. Heliyon.

[52] Cui F.J., Yang Y.M., Sun L., Zan X.Y., Sun W.J., Zeb U. (2024). Grifola frondosa polysaccharides: A review on structure/activity, biosynthesis and engineering strategies. Int. J. Biol. Macromol..

[53] Gravina A.G., Pellegrino R., Auletta S., Palladino G., Brandimarte G., D’Onofrio R., Arboretto G., Imperio G., Ventura A., Cipullo M. (2023). Hericium erinaceus, a medicinal fungus with a centuries-old history: Evidence in gastrointestinal diseases. World J. Gastroenterol..

[54] Sharma A., Bhardwaj G., Nayik G.A. (2023). Edible and Medicinal Mushrooms of the Himalayas.

[55] Pawlik A., Jaszek M., Stefaniuk D., Świderska-Burek U., Mazur A., Wielbo J., Koper P., Żebracki K., Janusz G. (2020). Combined effect of light and nutrients on the micromorphology of the white rot fungus Cerrena unicolor. Int. J. Mol. Sci..

[56] Bernicchia A., Gorjón S. (2020). Polypores of the Mediterranean Region.

[57] Michniewicz A., Ullrich R., Ledakowicz S., Hofrichter M. (2006). The white-rot fungus Cerrena unicolor strain 137 produces two laccase isoforms with different physico-chemical and catalytic properties. Appl. Microbiol. Biotechnol..

[58] Pawlik A., Ruminowicz-Stefaniuk M., Frac M., Mazur A., Wielbo J., Janusz G. (2019). The wood decay fungus Cerrena unicolor adjusts its metabolism to grow on various types of wood and light conditions. PLoS ONE.

[59] Belova O.V., Lisov A.V., Vinokurova N.G., Kostenevich A.A., Sapunova L.I., Lobanok A.G., Leont’evskiĭ A.A. (2014). [Xylanase and cellulase of fungus Cerrena unicolor VKM F-3196: Production, properties, and applications for the saccharification of plant material]. Prikl. Biokhim. Mikrobiol..

[60] Pawlik A., Ciołek B., Sulej J., Mazur A., Grela P., Staszczak M., Niścior M., Jaszek M., Matuszewska A., Janusz G. (2021). Cerrena unicolor Laccases, Genes Expression and Regulation of Activity. Biomolecules.

[61] Wang J., Liu K., Gong W., Wang Q., Xu D., Liu M., Bi K., Song Y. (2012). Anticancer, antioxidant, and antimicrobial activities of anemone (*Anemone cathayensis*). Food Sci. Biotechnol..

[62] Jaszek M., Osińska-Jaroszuk M., Janusz G., Matuszewska A., Stefaniuk D., Sulej J., Polak J., Ruminowicz M., Grzywnowicz K., Jarosz-Wilkołazka A. (2013). New bioactive fungal molecules with high antioxidant and antimicrobial capacity isolated from Cerrena unicolor idiophasic cultures. Biomed Res. Int..

[63] Zhang L.B., Yang W.W.J., Qiu T.T. (2023). Genome-wide study of *Cerrena unicolor* 87613 laccase gene family and their mode prediction in association with substrate oxidation. BMC Genom..

[64] Sevindik M. (2018). Antioxidant and antimicrobial activity of Cerrena unicolor. Mycopath.

[65] Matuszewska A., Jaszek M., Stefaniuk D., Ciszewski T., Matuszewski Ł. (2018). Anticancer, antioxidant, and antibacterial activities of low molecular weight bioactive subfractions isolated from cultures of wood degrading fungus Cerrena unicolor. PLoS ONE.

[66] Matuszewska A., Stefaniuk D., Jaszek M., Pięt M., Zając A., Matuszewski Ł., Cios I., Grąz M., Paduch R., Bancerz R. (2019). Antitumor potential of new low molecular weight antioxidative preparations from the white rot fungus Cerrena unicolor against human colon cancer cells. Sci. Rep..

[67] Pięt M., Zając A., Paduch R., Jaszek M., Frant M., Stefaniuk D., Matuszewska A., Grzywnowicz K. (2021). Chemopreventive activity of bioactive fungal fractions isolated from milk-supplemented cultures of *Cerrena unicolor* and *Pycnoporus sanguineus* on colon cancer cells. 3 Biotech.

[68] Fitzmaurice C., Allen C., Barber R.M., Barregard L., Bhutta Z.A., Brenner H., Dicker D.J., Chimed-Orchir O., Dandona R., Dandona L. (2017). Global, Regional, and National Cancer Incidence, Mortality, Years of Life Lost, Years Lived with Disability, and Disability-Adjusted Life-years for 32 Cancer Groups, 1990 to 2015: A Systematic Analysis for the Global Burden of Disease Study. JAMA Oncol..

[69] Zając A., Pięt M., Stefaniuk D., Chojnacki M., Jakubowicz-Gil J., Paduch R., Matuszewska A., Jaszek M. (2021). Pro-health and anti-cancer activity of fungal fractions isolated from milk-supplemented cultures of lentinus (Pleurotus) sajor-caju. Biomolecules.

[70] Joseph T.P., Chanda W., Padhiar A.A., Batool S., LiQun S., Zhong M., Huang M. (2018). A Preclinical Evaluation of the Antitumor Activities of Edible and Medicinal Mushrooms: A Molecular Insight. Integr. Cancer Ther..

[71] Zhang Y., Zhang M., Jiang Y., Li X., He Y., Zeng P., Guo Z., Chang Y., Luo H., Liu Y. (2018). Lentinan as an immunotherapeutic for treating lung cancer: A review of 12 years clinical studies in China. J. Cancer Res. Clin. Oncol..

[72] Matuszewska A., Karp M., Jaszek M., Janusz G., Osińska-Jaroszuk M., Sulej J., Stefaniuk D., Tomczak W., Giannopoulos K. (2016). Laccase purified from Cerrena unicolor exerts antitumor activity against leukemic cells. Oncol. Lett..

[73] Mizerska-Dudka M., Jaszek M., Błachowicz A., Rejczak T.P., Matuszewska A., Osińska-Jaroszuk M., Stefaniuk D., Janusz G., Sulej J., Kandefer-Szerszeń M. (2015). Fungus Cerrena unicolor as an effective source of new antiviral, immunomodulatory, and anticancer compounds. Int. J. Biol. Macromol..

[74] Prendecka-Wróbel M., Pigoń-Zając D., Jaszek M., Matuszewska A., Stefaniuk D., Opielak G., Piotrowska K., Rahnama-Hezavah M., Małecka-Massalska T. (2022). Electric Cell-Substrate Impedance Sensing (ECIS) as a Convenient Tool to Assess the Potential of Low Molecular Fraction Derived from Medicinal Fungus *Cerrena unicolor* in Action on L929 and CT-26 Cell Lines. Molecules.

[75] Pigoń-Zając D., Derlatka K., Chuchmacz W., Kulczycka M., Grzelczak K., Stefaniuk D., Matuszewska A., Homa-Mlak I., Prendecka-Wróbel M., Małecka-Massalska T. (2024). The anticancer activity of laccase from white rot fungus Cerrena unicolor on the example of its action on Caov-3 and NIH:OVCAR-3 ovarian cancer cells. Ann. Agric. Environ. Med..

[76] Sondej D., Pigoń-Zając D., Jaszek M., Stefaniuk D., Matuszewska A., Bielak K., Opielak G., Małecka-Massalska T., Rahnama-Hezavah M., Prendecka-Wróbel M. (2025). Is laccase from medicinal mushroom Cerrena unicolor cytotoxic to colon cancer cell line CT-26?. PLoS ONE.

[77] Chaiharn M., Phutdhawong W.S., Amornlerdpison D., Phutdhawong W. (2018). Antibacterial, antioxidant properties and bioactive compounds of thai cultivated mushroom extracts against food-borne bacterial strains. Chiang Mai J. Sci..

[78] Demir M.S., Yamac M. (2008). Antimicrobial activities of basidiocarp, submerged mycelium and exopolysaccharide of some native Basidiomycetes strains. J. Appl. Biol. Sci..

[79] Fong D., Chan M.M. (2022). Soil-Transmitted Helminth Infections. Human Parasites.

[80] Ziaja-Sołtys M., Kołodziej P., Stefaniuk D., Matuszewska A., Jaszek M., Bogucka-Kocka A. (2022). Low-Molecular-Weight Secondary Metabolites from Fungi: Cerrena unicolor as a New Proposal of an Effective Preparation against Rhabditis Nematodes. Molecules.

[81] Uyanoglu M., Yamac M., Canbek M., Senturk H., Kartkaya K., Oglakci A., Turgak O., Kanbak G. (2013). Curative effect of crude exopolysaccharides of some macrofungi on alcohol-induced liver damage. Ultrastruct. Pathol..

[82] Zhou Z., Li R., Ng T.B., Lai Y., Yang J., Ye X. (2020). A New Laccase of Lac 2 from the White Rot Fungus Cerrena unicolor 6884 and Lac 2-Mediated Degradation of Aflatoxin B(1). Toxins.

[83] Yamaç M., Zeytinoglu M., Kanbak G., Bayramoglu G., Senturk H. (2009). Hypoglycemic effect of crude exopolysaccharides produced by Cerrena unicolor, Coprinus comatus, and Lenzites betulina isolates in streptozotocin- induced diabetic rats. Pharm. Biol..

[84] Deng X., Gould M., Ali M.A. (2022). A review of current advancements for wound healing: Biomaterial applications and medical devices. J. Biomed. Mater. Res. Part B Appl. Biomater..

[85] Sharifi-Rad J., Butnariu M., Ezzat S.M., Adetunji C.O., Imran M., Sobhani S.R., Tufail T., Hosseinabadi T., Ramírez-Alarcón K., Martorell M. (2020). Mushrooms-Rich Preparations on Wound Healing: From Nutritional to Medicinal Attributes. Front. Pharmacol..

[86] Stefaniuk D., Misztal T., Pięt M., Zając A., Kopycińska M., Matuszewska A., Ruminowicz-Stefaniuk M., Matuszewski Ł., Marcińczyk N., Belcarz A. (2021). Thromboelastometric analysis of anticancer cerrena unicolor subfractions reveal their potential as fibrin glue drug carrier enhancers. Biomolecules.

[87] Berovic M., Podgornik B.B., Gregori A. (2022). Cultivation Technologies for Production of Medicinal Mushroom Biomass: Review. Int. J. Med. Mushrooms.

